# Intra- and supra­molecular inter­actions in *cis*,*mer*-diaqua­tris­(1*H*-imidazole-κ*N* ^3^)(terephthalato-κ*O*)cobalt(II) monohydrate

**DOI:** 10.1107/S1600536812011993

**Published:** 2012-03-24

**Authors:** Aouaouche Benkanoun, Fadila Balegroune, Achoura Guehria-Laïdoudi, Slimane Dahaoui, Claude Lecomte

**Affiliations:** a Laboratoire de Cristallographie-Thermodynamique, Faculté de Chimie, U.S.T.H.B., BP 32 El-Alia, Bab-Ezzouar 16111, Alger, Algeria; b CRM 2,CNRS-UPRESA 7036, Université Henry Poincaré, Faculté des Sciences et Techniques, BP 70239, 54506 Vandoeuvres, Les Nancy Cedex, France

## Abstract

In the title compound, [Co(C_8_H_4_O_4_)(C_3_H_4_N_2_)_3_(H_2_O)_2_]·H_2_O, the *cisoid* angles are in the range 85.59 (5)–93.56 (5)°, while two equal *transoid* angles deviate significantly from the ideal linear angle, the third being almost linear. One carboxyl­ate group is almost coplanar [1.23 (13)°] with the plane of its parent aromatic ring, although it has one O-atom donor involved in one coordination and one hydrogen bond as acceptor. The other carboxyl­ate group does not coordinate and is rotated out of this plane with a torsional twist of 17.27 (20)°. The coordination neutral entity, based on aqua ligands and two cyclic co-ligands seems, at first sight, monomeric. Strongly tight, *via* one intra­molecular hydrogen bond between aqua and carboxyl­ate O atoms, it brings out a quasi-planar six-membered ring around the Co^II^ atom, turning the CoN_3_O_3_ coordination octa­hedron into a new building block. The rigidity of this feature associated with several hydrogen-bonded arrays yields an extended structure. In the resulting supra­molecular packing, a binuclear hydrated Co^II^ assembly, built up from triple strands driven by different heterosynthons, embodies the synergy of coordination, covalent and hydrogen bonds.

## Related literature

For general background to important structural features inducing some inter­esting properties, see: Chen *et al.* (1996 ▶); Yang *et al.* (2002 ▶); Ye & Chen (2003 ▶); Xie *et al.* (2009 ▶); Baca *et al.* (2003 ▶). For related compounds or structures, see: Niu *et al.* (2004 ▶); Tong *et al.* (2002 ▶); Liu *et al.* (2001 ▶, 2003 ▶); Zeng *et al.* (1997 ▶).
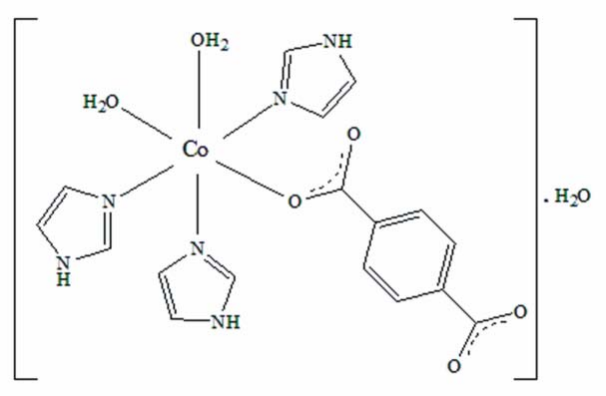


## Experimental

### 

#### Crystal data

[Co(C_8_H_4_O_4_)(C_3_H_4_N_2_)_3_(H_2_O)_2_]·H_2_O
*M* *_r_* = 481.34
Monoclinic, 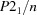

*a* = 7.65363 (8) Å
*b* = 10.45169 (13) Å
*c* = 24.7538 (3) Å
β = 90.227 (1)°
*V* = 1980.12 (4) Å^3^
*Z* = 4
Mo *K*α radiation
μ = 0.92 mm^−1^
*T* = 291 K
0.21 × 0.14 × 0.08 mm

#### Data collection

Oxford Diffraction Xcalibur diffractometer
Absorption correction: analytical [*CrysAlis RED* (Oxford Diffraction, 2009 ▶), using a multi-faceted crystal model based on expressions derived by Clark & Reid (1995 ▶)] *T*_min_ = 0.871, *T*_max_ = 0.935
86135 measured reflections
4760 independent reflections
4153 reflections with *I* > 2σ(*I*)
*R*_int_ = 0.066

#### Refinement

*R*[*F* ^2^ > 2σ(*F* ^2^)] = 0.029
*wR*(*F* ^2^) = 0.071
*S* = 1.08
4760 reflections
316 parameters
H atoms treated by a mixture of independent and constrained refinement
Δρ_max_ = 0.40 e Å^−3^
Δρ_min_ = −0.33 e Å^−3^

### 

Data collection: *CrysAlis CCD* (Oxford Diffraction, 2009 ▶); cell refinement: *CrysAlis RED* (Oxford Diffraction, 2009 ▶); data reduction: *CrysAlis RED*; program(s) used to solve structure: *SHELXS97* (Sheldrick, 2008 ▶); program(s) used to refine structure: *SHELXL97* (Sheldrick, 2008 ▶); molecular graphics: *ORTEP-3 for Windows* (Farrugia, 1997 ▶); software used to prepare material for publication: *WinGX* (Farrugia, 1999 ▶).

## Supplementary Material

Crystal structure: contains datablock(s) global, I. DOI: 10.1107/S1600536812011993/ds2181sup1.cif

Structure factors: contains datablock(s) I. DOI: 10.1107/S1600536812011993/ds2181Isup2.hkl

Additional supplementary materials:  crystallographic information; 3D view; checkCIF report

## Figures and Tables

**Table 1:** Selected geometric parameters (Å, °)

Co—O2*W*	2.1064 (11)
Co—N3	2.1076 (13)
Co—N5	2.1124 (13)
Co—N1	2.1347 (13)
Co—O1	2.1442 (10)
Co—O1*W*	2.1680 (11)

**Table 2:** Hydrogen-bond geometry (Å, °)

*D*—H⋯*A*	*D*—H	H⋯*A*	*D*⋯*A*	*D*—H⋯*A*
N2—H2*N*⋯O1^i^	0.86 (2)	2.09 (2)	2.9204 (17)	162 (2)
N4—H4*N*⋯O3*W*^ii^	0.85 (2)	2.18 (2)	2.9842 (18)	157.0 (19)
N4—H4*N*⋯O4^iii^	0.85 (2)	2.50 (2)	2.9521 (18)	114.5 (17)
N6—H6*N*⋯O3^iv^	0.84 (2)	2.02 (2)	2.8249 (18)	162 (2)
O1*W*—H1*W*⋯O2	0.85 (3)	1.79 (3)	2.6160 (16)	163 (3)
O1*W*—H2*W*⋯O4^v^	0.85 (3)	1.85 (3)	2.6606 (16)	161 (2)
O2*W*—H3*W*⋯O3^vi^	0.82 (3)	1.95 (3)	2.7516 (16)	168 (2)
O2*W*—H4*W*⋯O3*W*^vii^	0.82 (2)	2.00 (2)	2.8118 (17)	172 (2)
O3*W*—H5*W*⋯O1*W*	0.81 (2)	2.08 (2)	2.8632 (16)	164 (2)
O3*W*—H6*W*⋯O3^vi^	0.82 (3)	1.95 (3)	2.7528 (17)	167 (2)

Symmetry codes: (i) 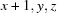; (ii) 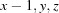; (iii) 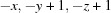; (iv) 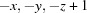; (v) 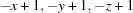; (vi) 
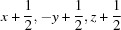
; (vii) 
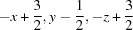
.

**Fig. 1.**
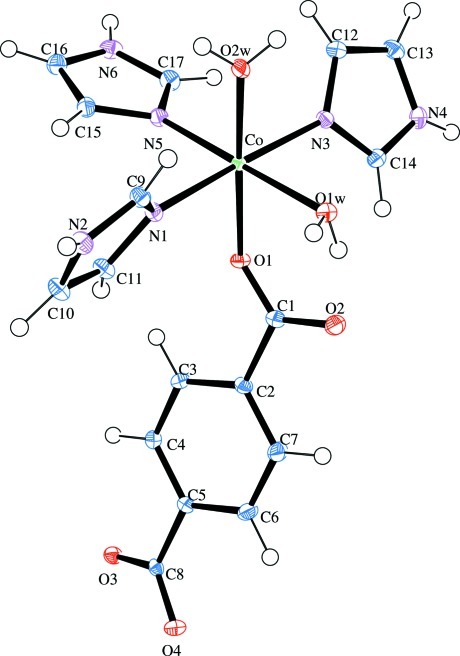
: The molecular structure of the title compound with displacement ellipsoids drawn at the 50% probability level.

**Fig. 2.**
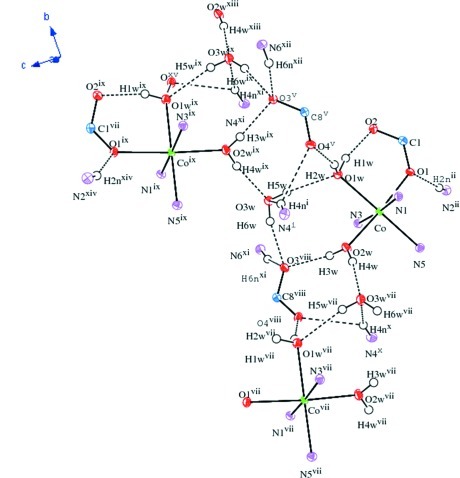
: Helical hydrogen-bonded ladder composed of R33(8) and R44(10). Symmetry codes: i: x + 1,y,z; ii: x - 1,y,z; v: -x + 1,-y + 1,-z + 1; vii: -x + 3/2,y - 1/2,-z + 3/2; viii: x - 1/2,-y - 1/2,-z - 1/2; ix: -x + 3/2,y + 1/2,-z + 3/2; x: -x + 1/2,y - 1/2,-z + 3/2 xi: -x + 1/2,y + 1/2,-z + 3/2, xii: x + 1, y + 1,z; xiii: x,y + 1,z; xiv: -x + 5/2,y + 1/2,-z + 3/2, xv: x - 1/2,-y + 1/2,z - 1/2

**Fig. 3.**
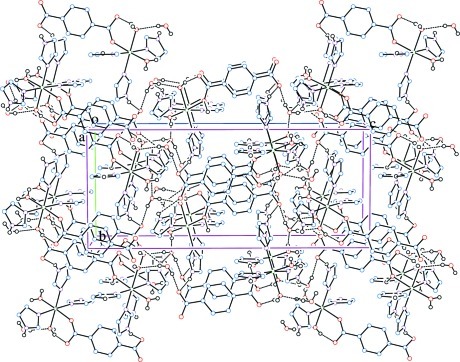
: Packing diagram viewed along [100] showing hydrogen bonds and ladder's layers.

**Fig. 4.**
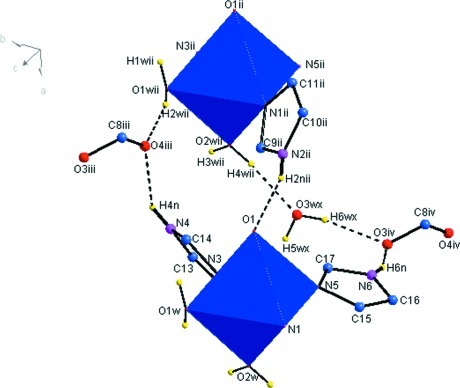
: Binuclear CoII assembly with triple strand. Symmetry codes: ii: x - 1,y,z; iii: -x,-y + 1,-z + 1; iv: -x,-y,-z + 1; x: -x + 1/2,y - 1/2,-z + 3/2

## supplementary crystallographic information

### Comment

This Cobalt-based compound, is the first isomer evidenced, with two aqua ligands *cis* and three N-coordinated imidazole ligands meridional, relative to the Co^II^ centre (Fig.1). Although it is built up from monomeric entity, it exhibits a packing with a relatively interesting metal-metal separation. As shown in Table 1, the Co-Ocarboxylate and the average Co—Ow distances are very closed and are comparable to those observed in related compounds (Niu *et al.*, 2004; Tong *et al.*, 2002). The three independent imidazole groups, and the single terephthalate dianion, both unidentate, participate respectively as donor and acceptor, in strong to moderate hydrogen bonds, and allow the recognition of supramolecular dimensionality (Table 2). The backbone of the architecture is the helical hydrogen-bonded ladder running along *b* axis, composed of alternating *R*^4^_4_(10) and *R*^3^_3_(8) heterosynthons (Fig.2), which are developed in turn, in bicyclic sheets, both of them containing a six-membered ring, and connected to imidazole ligands *via* secondary N atom. The former is associated with a crossed *R*^2^_2_(6) and involves one bifurcated intermolecular H-bond. The latter shares a common Co—*O*(aqua) bond with a cycle formed of two coordination and three covalent bondings, beside one intramolecular H-bond (Table 2). The resulting quasi-planar six-membered ring, is responsible of environment's rigidity around the metal centre and changes the coordination octahedron in a new building block. The three imidazoles participate as donor with oxygen atom of adjacent carboxylates, and they bring about polymeric chain which mimics the carboxylate-histidine-zinc triad systems (Chen *et al.*, 1996; Yang *et al.*, 2002; Ye *et al.*, 2003; Xie *et al.*, 2009). As seen in Fig.2 and Fig.3, all these overlapping subnetworks lead to the formation of cross-linked supramolecular layers where additional single H-bonds provided by secondary N atom of the imidazoles in one hand, and the spacer of the dicarboxylate on other hand, achieve a well organized three-dimensional packing. Within this three-dimensional framework, the binuclear Co^II^ assembly showing the shortest and probably interesting (Baca *et al.*, 2003) separation metal-metal of 7.6536 (1) Å is built up from triple strand driven by *R*^3^_3_(14), *R*^4^_4_(16) and *R*^5^_5_(18) heterosynthons (Fig.4). A comparison with the two chemically similar Co^II^ compounds [Co(C_3_H_4_N_2_)_4_(H_2_O)_2_](C_8_H_4_0_4_), and [Co(C_3_H_4_N_2_)](C_8_H_4_0_4_).4H_2_O (Tong *et al.*, 2002), as well as their isostructural compounds obtained with Mn(II) (Liu *et al.*, 2001;2003) and Cu(II) (Zeng *et al.*, 1997), reveals that their building blocks are mononuclear, and the terephthalate dianion doesn't get involved in coordination. With this study, we may confirm that in this structure, a competition takes place between terephthalate and water and it is probably the presence of both coordinated and uncoordinated water molecules, which builts a new building block, by enhancing the dicarboxylato ligand ability to get involved in coordination sphere.

### Experimental

A mixture of CoCl_2_,6H_2_O (0.24 g, 1 mmol), terephthalic acid (0.17 g,1 mmol), imidazole (0.14 g, 2 mmol) NaOH (0.08 g, 2 mmol) and water (15 ml) was stirred for 30 min at room temperature, then transferred in a 25 ml Teflon-lined stainless steel reactor, then sealed and heated at 120°C for 72 h. Upon cooling to room temperature, light-pink crystals of title compound suitable for X-ray crystallographic analysis were obtained.

### Refinement

H atoms attached to C atoms were positioned at calculated positions and were treated as riding on the parent atoms, with C—H=0.95 Å and *U*_iso_(H)=1.2Ueq(C). Water hydrogen atoms and H atoms bonded to N atoms were located in a difference map and refined isotropically.

### Crystal data

**Table:**

[Co(C_8_H_4_O_4_)(C_3_H_4_N_2_)_3_(H_2_O)_2_]·H_2_O	*F*(000) = 996
*M**_r_* = 481.34	*D*_x_ = 1.615 Mg m^−^^3^
Monoclinic, *P*2_1_/*n*	Mo *K*α radiation, λ = 0.71073 Å
Hall symbol: -P 2yn	Cell parameters from 35056 reflections
*a* = 7.65363 (8) Å	θ = 3.3–32.9°
*b* = 10.45169 (13) Å	µ = 0.92 mm^−^^1^
*c* = 24.7538 (3) Å	*T* = 291 K
β = 90.227 (1)°	Prism, pink
*V* = 1980.12 (4) Å^3^	0.21 × 0.14 × 0.08 mm
*Z* = 4	

### Data collection

**Table:**

Oxford Diffraction Xcalibur diffractometer	4760 independent reflections
Radiation source: fine-focus sealed tube	4153 reflections with *I* > 2σ(*I*)
Graphite monochromator	*R*_int_ = 0.066
Detector resolution: 10.4508 pixels mm^-1^	θ_max_ = 28.0°, θ_min_ = 3.4°
wσcans	*h* = −10→10
Absorption correction: analytical [*CrysAlis RED* (Oxford Diffraction, 2009), using a multi-faceted crystal model based on expressions derived by Clark & Reid (1995)]	*k* = −13→13
*T*_min_ = 0.871, *T*_max_ = 0.935	*l* = −32→32
86135 measured reflections	

### Refinement

**Table:**

Refinement on *F*^2^	Primary atom site location: structure-invariant direct methods
Least-squares matrix: full	Secondary atom site location: difference Fourier map
*R*[*F*^2^ > 2σ(*F*^2^)] = 0.029	Hydrogen site location: inferred from neighbouring sites
*wR*(*F*^2^) = 0.071	H atoms treated by a mixture of independent and constrained refinement
*S* = 1.08	*w* = 1/[σ^2^(*F*_o_^2^) + (0.0265*P*)^2^ + 1.5429*P*] where *P* = (*F*_o_^2^ + 2*F*_c_^2^)/3
4760 reflections	(Δ/σ)_max_ = 0.003
316 parameters	Δρ_max_ = 0.40 e Å^−^^3^
0 restraints	Δρ_min_ = −0.33 e Å^−^^3^

### Special details

**Table:**

Experimental. Absorption correction: CrysAlis RED, Oxford Diffraction (2009) Analytical numerical absorption correction using a multifaceted crystal model based on expressions derived by Clark & Reid (1995).
Geometry. All e.s.d.'s (except the e.s.d. in the dihedral angle between two l.s. planes) are estimated using the full covariance matrix. The cell e.s.d.'s are taken into account individually in the estimation of e.s.d.'s in distances, angles and torsion angles; correlations between e.s.d.'s in cell parameters are only used when they are defined by crystal symmetry. An approximate (isotropic) treatment of cell e.s.d.'s is used for estimating e.s.d.'s involving l.s. planes.
Refinement. *CrysAlis RED*, Oxford Diffraction Ltd Refinement of *F*^2^ against ALL reflections. The weighted *R*-factor *wR* and goodness of fit *S* are based on *F*^2^, conventional *R*-factors *R* are based on *F*, with *F* set to zero for negative *F*^2^. The threshold expression of *F*^2^ > σ(*F*^2^) is used only for calculating *R*-factors(gt) *etc*. and is not relevant to the choice of reflections for refinement. *R*-factors based on *F*^2^ are statistically about twice as large as those based on *F*, and *R*- factors based on ALL data will be even larger.

### Fractional atomic coordinates and isotropic or equivalent isotropic displacement parameters (Å^2^)

**Table:**

	*x*	*y*	*z*	*U*_iso_*/*U*_eq_	
Co	0.44254 (2)	0.144772 (19)	0.652633 (8)	0.00874 (6)	
O1W	0.52240 (15)	0.33528 (11)	0.67705 (4)	0.0123 (2)	
O2W	0.60355 (16)	0.07515 (12)	0.71527 (5)	0.0147 (2)	
O3W	0.66608 (15)	0.36561 (12)	0.78325 (5)	0.0149 (2)	
O1	0.29257 (14)	0.22971 (10)	0.58868 (4)	0.0115 (2)	
O2	0.31457 (15)	0.43979 (11)	0.60544 (4)	0.0165 (2)	
O3	0.05014 (14)	0.36582 (10)	0.32226 (4)	0.0130 (2)	
O4	0.16128 (14)	0.56111 (11)	0.33113 (4)	0.0134 (2)	
C1	0.28701 (19)	0.34725 (15)	0.57476 (6)	0.0116 (3)	
C2	0.24336 (19)	0.37422 (15)	0.51627 (6)	0.0115 (3)	
C3	0.21121 (19)	0.27458 (15)	0.48037 (6)	0.0115 (3)	
H3	0.2188	0.1885	0.4925	0.014*	
C4	0.16812 (19)	0.30000 (15)	0.42695 (6)	0.0117 (3)	
H4	0.1412	0.2315	0.4031	0.014*	
C5	0.16416 (19)	0.42578 (15)	0.40819 (6)	0.0106 (3)	
C6	0.2013 (2)	0.52539 (15)	0.44372 (6)	0.0154 (3)	
H6	0.2019	0.6111	0.4310	0.018*	
C7	0.2375 (2)	0.49999 (16)	0.49776 (6)	0.0160 (3)	
H7	0.2584	0.5686	0.5221	0.019*	
C8	0.12281 (19)	0.45417 (14)	0.34977 (6)	0.0101 (3)	
N1	0.66337 (16)	0.13362 (12)	0.60043 (5)	0.0113 (3)	
N2	0.93656 (18)	0.13617 (13)	0.57266 (6)	0.0143 (3)	
C9	0.8290 (2)	0.13727 (15)	0.61550 (6)	0.0128 (3)	
H9	0.8674	0.1403	0.6520	0.015*	
C10	0.8336 (2)	0.13227 (16)	0.52700 (6)	0.0155 (3)	
H10	0.8722	0.1310	0.4906	0.019*	
C11	0.6653 (2)	0.13062 (15)	0.54460 (6)	0.0143 (3)	
H11	0.5647	0.1278	0.5220	0.017*	
N3	0.23535 (16)	0.16873 (12)	0.70755 (5)	0.0115 (3)	
N4	−0.00080 (18)	0.25190 (14)	0.74341 (5)	0.0145 (3)	
C12	0.1779 (2)	0.09015 (16)	0.74877 (6)	0.0146 (3)	
H12	0.2327	0.0129	0.7599	0.017*	
C13	0.0312 (2)	0.14045 (16)	0.77079 (6)	0.0152 (3)	
H13	−0.0359	0.1054	0.7994	0.018*	
C14	0.12406 (19)	0.26542 (15)	0.70594 (6)	0.0127 (3)	
H14	0.1315	0.3352	0.6815	0.015*	
N5	0.36196 (16)	−0.04089 (12)	0.62981 (5)	0.0120 (3)	
N6	0.23014 (18)	−0.22776 (14)	0.63208 (6)	0.0157 (3)	
C15	0.4649 (2)	−0.13139 (15)	0.60470 (6)	0.0141 (3)	
H15	0.5754	−0.1150	0.5888	0.017*	
C16	0.3850 (2)	−0.24738 (16)	0.60607 (6)	0.0159 (3)	
H16	0.4278	−0.3258	0.5919	0.019*	
C17	0.2212 (2)	−0.10428 (16)	0.64582 (6)	0.0147 (3)	
H17	0.1262	−0.0667	0.6647	0.018*	
H2N	1.048 (3)	0.147 (2)	0.5743 (9)	0.030 (6)*	
H4N	−0.088 (3)	0.301 (2)	0.7480 (8)	0.022 (5)*	
H6N	0.156 (3)	−0.283 (2)	0.6416 (9)	0.026 (6)*	
H1W	0.454 (3)	0.381 (3)	0.6584 (10)	0.043 (7)*	
H2W	0.623 (3)	0.360 (2)	0.6673 (9)	0.034 (6)*	
H3W	0.576 (3)	0.085 (2)	0.7469 (10)	0.036 (7)*	
H4W	0.670 (3)	0.014 (2)	0.7123 (9)	0.029 (6)*	
H5W	0.610 (3)	0.365 (2)	0.7556 (9)	0.025 (6)*	
H6W	0.639 (3)	0.301 (2)	0.7995 (9)	0.031 (6)*	

### Atomic displacement parameters (Å^2^)

**Table:**

	*U*^11^	*U*^22^	*U*^33^	*U*^12^	*U*^13^	*U*^23^
Co	0.00883 (10)	0.00915 (11)	0.00823 (10)	−0.00041 (7)	0.00022 (7)	0.00042 (7)
O1W	0.0120 (5)	0.0125 (6)	0.0125 (5)	−0.0014 (4)	−0.0014 (4)	0.0007 (4)
O2W	0.0183 (6)	0.0166 (6)	0.0093 (5)	0.0060 (5)	−0.0001 (4)	0.0001 (4)
O3W	0.0167 (6)	0.0158 (6)	0.0120 (5)	−0.0031 (5)	−0.0020 (5)	0.0025 (5)
O1	0.0127 (5)	0.0111 (5)	0.0107 (5)	−0.0006 (4)	−0.0015 (4)	0.0024 (4)
O2	0.0244 (6)	0.0126 (5)	0.0125 (5)	−0.0002 (5)	−0.0050 (5)	−0.0009 (4)
O3	0.0158 (5)	0.0130 (5)	0.0101 (5)	−0.0024 (4)	−0.0015 (4)	−0.0003 (4)
O4	0.0146 (5)	0.0124 (5)	0.0134 (5)	−0.0022 (4)	−0.0017 (4)	0.0038 (4)
C1	0.0094 (6)	0.0142 (7)	0.0113 (7)	−0.0001 (6)	0.0001 (5)	0.0019 (6)
C2	0.0114 (7)	0.0137 (7)	0.0093 (7)	0.0006 (6)	−0.0007 (5)	0.0011 (6)
C3	0.0116 (7)	0.0103 (7)	0.0127 (7)	0.0001 (5)	−0.0008 (6)	0.0022 (6)
C4	0.0113 (7)	0.0116 (7)	0.0121 (7)	0.0003 (6)	0.0005 (5)	−0.0013 (6)
C5	0.0095 (6)	0.0125 (7)	0.0098 (7)	−0.0001 (5)	0.0000 (5)	0.0006 (6)
C6	0.0218 (8)	0.0101 (7)	0.0141 (7)	−0.0013 (6)	−0.0020 (6)	0.0015 (6)
C7	0.0230 (8)	0.0117 (8)	0.0131 (7)	−0.0015 (6)	−0.0037 (6)	−0.0015 (6)
C8	0.0079 (6)	0.0130 (7)	0.0095 (7)	0.0012 (5)	0.0011 (5)	−0.0006 (6)
N1	0.0120 (6)	0.0118 (6)	0.0101 (6)	−0.0012 (5)	0.0007 (5)	−0.0010 (5)
N2	0.0108 (6)	0.0168 (7)	0.0153 (7)	−0.0009 (5)	0.0021 (5)	0.0002 (5)
C9	0.0120 (7)	0.0138 (7)	0.0126 (7)	−0.0012 (6)	0.0009 (6)	0.0000 (6)
C10	0.0157 (7)	0.0194 (8)	0.0114 (7)	−0.0020 (6)	0.0029 (6)	−0.0007 (6)
C11	0.0153 (7)	0.0173 (8)	0.0105 (7)	−0.0021 (6)	0.0004 (6)	0.0002 (6)
N3	0.0116 (6)	0.0126 (6)	0.0104 (6)	0.0004 (5)	0.0008 (5)	0.0002 (5)
N4	0.0121 (6)	0.0157 (7)	0.0158 (7)	0.0017 (5)	0.0021 (5)	−0.0027 (5)
C12	0.0169 (7)	0.0143 (8)	0.0124 (7)	0.0000 (6)	0.0009 (6)	0.0024 (6)
C13	0.0151 (7)	0.0182 (8)	0.0123 (7)	−0.0036 (6)	0.0023 (6)	0.0010 (6)
C14	0.0127 (7)	0.0118 (7)	0.0135 (7)	0.0002 (6)	−0.0009 (6)	−0.0001 (6)
N5	0.0121 (6)	0.0115 (6)	0.0124 (6)	−0.0016 (5)	0.0011 (5)	−0.0002 (5)
N6	0.0172 (7)	0.0139 (7)	0.0161 (7)	−0.0062 (6)	−0.0014 (5)	0.0019 (5)
C15	0.0156 (7)	0.0131 (7)	0.0135 (7)	−0.0001 (6)	0.0019 (6)	−0.0009 (6)
C16	0.0200 (8)	0.0130 (8)	0.0147 (7)	−0.0010 (6)	−0.0015 (6)	−0.0014 (6)
C17	0.0131 (7)	0.0158 (8)	0.0151 (7)	−0.0023 (6)	0.0008 (6)	−0.0004 (6)

### Geometric parameters (Å, º)

**Table:**

Co—O2W	2.1064 (11)	N1—C9	1.3204 (19)
Co—N3	2.1076 (13)	N1—C11	1.3825 (19)
Co—N5	2.1124 (13)	N2—C9	1.345 (2)
Co—N1	2.1347 (13)	N2—C10	1.376 (2)
Co—O1	2.1442 (10)	N2—H2N	0.86 (2)
Co—O1W	2.1680 (11)	C9—H9	0.9500
O1W—H1W	0.85 (3)	C10—C11	1.362 (2)
O1W—H2W	0.85 (3)	C10—H10	0.9500
O2W—H3W	0.82 (3)	C11—H11	0.9500
O2W—H4W	0.82 (2)	N3—C14	1.322 (2)
O3W—H5W	0.81 (2)	N3—C12	1.383 (2)
O3W—H6W	0.82 (3)	N4—C14	1.342 (2)
O1—C1	1.2765 (19)	N4—C13	1.369 (2)
O2—C1	1.2471 (19)	N4—H4N	0.85 (2)
O3—C8	1.2738 (18)	C12—C13	1.356 (2)
O4—C8	1.2450 (19)	C12—H12	0.9500
C1—C2	1.511 (2)	C13—H13	0.9500
C2—C3	1.390 (2)	C14—H14	0.9500
C2—C7	1.393 (2)	N5—C17	1.327 (2)
C3—C4	1.387 (2)	N5—C15	1.380 (2)
C3—H3	0.9500	N6—C17	1.337 (2)
C4—C5	1.395 (2)	N6—C16	1.366 (2)
C4—H4	0.9500	N6—H6N	0.84 (2)
C5—C6	1.391 (2)	C15—C16	1.358 (2)
C5—C8	1.509 (2)	C15—H15	0.9500
C6—C7	1.391 (2)	C16—H16	0.9500
C6—H6	0.9500	C17—H17	0.9500
C7—H7	0.9500		
			
O2W—Co—N3	90.33 (5)	O3—C8—C5	117.34 (13)
O2W—Co—N5	92.80 (5)	C9—N1—C11	105.60 (13)
N3—Co—N5	93.56 (5)	C9—N1—Co	126.08 (10)
O2W—Co—N1	87.96 (5)	C11—N1—Co	128.17 (10)
N3—Co—N1	175.12 (5)	C9—N2—C10	107.31 (13)
N5—Co—N1	91.09 (5)	C9—N2—H2N	124.9 (15)
O2W—Co—O1	175.14 (5)	C10—N2—H2N	127.3 (15)
N3—Co—O1	91.44 (4)	N1—C9—N2	111.54 (14)
N5—Co—O1	91.60 (5)	N1—C9—H9	124.2
N1—Co—O1	89.91 (5)	N2—C9—H9	124.2
O2W—Co—O1W	87.05 (5)	C11—C10—N2	106.07 (14)
N3—Co—O1W	85.59 (5)	C11—C10—H10	127.0
N5—Co—O1W	179.13 (5)	N2—C10—H10	127.0
N1—Co—O1W	89.76 (5)	C10—C11—N1	109.48 (14)
O1—Co—O1W	88.57 (4)	C10—C11—H11	125.3
Co—O1W—H1W	101.0 (18)	N1—C11—H11	125.3
Co—O1W—H2W	117.2 (16)	C14—N3—C12	105.64 (13)
H1W—O1W—H2W	104 (2)	C14—N3—Co	123.89 (11)
Co—O2W—H3W	120.8 (17)	C12—N3—Co	130.35 (11)
Co—O2W—H4W	124.3 (16)	C14—N4—C13	107.78 (14)
H3W—O2W—H4W	110 (2)	C14—N4—H4N	126.4 (14)
H5W—O3W—H6W	106 (2)	C13—N4—H4N	125.7 (14)
C1—O1—Co	127.94 (10)	C13—C12—N3	109.42 (14)
O2—C1—O1	125.22 (14)	C13—C12—H12	125.3
O2—C1—C2	118.35 (14)	N3—C12—H12	125.3
O1—C1—C2	116.43 (13)	C12—C13—N4	106.11 (14)
C3—C2—C7	119.42 (14)	C12—C13—H13	126.9
C3—C2—C1	120.70 (14)	N4—C13—H13	126.9
C7—C2—C1	119.87 (14)	N3—C14—N4	111.05 (14)
C4—C3—C2	120.44 (14)	N3—C14—H14	124.5
C4—C3—H3	119.8	N4—C14—H14	124.5
C2—C3—H3	119.8	C17—N5—C15	104.94 (13)
C3—C4—C5	120.18 (14)	C17—N5—Co	127.98 (11)
C3—C4—H4	119.9	C15—N5—Co	125.69 (10)
C5—C4—H4	119.9	C17—N6—C16	108.07 (14)
C6—C5—C4	119.39 (14)	C17—N6—H6N	123.5 (15)
C6—C5—C8	120.01 (14)	C16—N6—H6N	128.1 (15)
C4—C5—C8	120.59 (13)	C16—C15—N5	110.03 (14)
C7—C6—C5	120.30 (15)	C16—C15—H15	125.0
C7—C6—H6	119.9	N5—C15—H15	125.0
C5—C6—H6	119.9	C15—C16—N6	105.63 (14)
C6—C7—C2	120.18 (15)	C15—C16—H16	127.2
C6—C7—H7	119.9	N6—C16—H16	127.2
C2—C7—H7	119.9	N5—C17—N6	111.33 (14)
O4—C8—O3	123.78 (14)	N5—C17—H17	124.3
O4—C8—C5	118.87 (13)	N6—C17—H17	124.3

### Hydrogen-bond geometry (Å, º)

**Table:**

*D*—H···*A*	*D*—H	H···*A*	*D*···*A*	*D*—H···*A*
N2—H2*N*···O1^i^	0.86 (2)	2.09 (2)	2.9204 (17)	162 (2)
N4—H4*N*···O3*W*^ii^	0.85 (2)	2.18 (2)	2.9842 (18)	157.0 (19)
N4—H4*N*···O4^iii^	0.85 (2)	2.50 (2)	2.9521 (18)	114.5 (17)
N6—H6*N*···O3^iv^	0.84 (2)	2.02 (2)	2.8249 (18)	162 (2)
O1*W*—H1*W*···O2	0.85 (3)	1.79 (3)	2.6160 (16)	163 (3)
O1*W*—H2*W*···O4^v^	0.85 (3)	1.85 (3)	2.6606 (16)	161 (2)
O2*W*—H3*W*···O3^vi^	0.82 (3)	1.95 (3)	2.7516 (16)	168 (2)
O2*W*—H4*W*···O3*W*^vii^	0.82 (2)	2.00 (2)	2.8118 (17)	172 (2)
O3*W*—H5*W*···O1*W*	0.81 (2)	2.08 (2)	2.8632 (16)	164 (2)
O3*W*—H6*W*···O3^vi^	0.82 (3)	1.95 (3)	2.7528 (17)	167 (2)

Symmetry codes: (i) *x*+1, *y*, *z*; (ii) *x*−1, *y*, *z*; (iii) −*x*, −*y*+1, −*z*+1; (iv) −*x*, −*y*, −*z*+1; (v) −*x*+1, −*y*+1, −*z*+1; (vi) *x*+1/2, −*y*+1/2, *z*+1/2; (vii) −*x*+3/2, *y*−1/2, −*z*+3/2.
